# Preserving cultural heritage through the valorization of Cordillera heirloom rice in the Philippines

**DOI:** 10.1007/s10460-020-10159-w

**Published:** 2020-10-15

**Authors:** Subir Bairagi, Marie Claire Custodio, Alvaro Durand-Morat, Matty Demont

**Affiliations:** 1 Department of Agricultural Economics and Agribusiness, University of Arkansas, Fayetteville, AR 72701 USA; 2 Agri-food Policy Platform, International Rice Research Institute (IRRI), 4031 Los Baños, Laguna Philippines

**Keywords:** Heritage farming, Heirloom crops, Landraces, Biodiversity, Survey, Willingness to pay

## Abstract

For centuries, heirloom rice varieties have been grown on the terraces of the Cordillera Mountains of Luzon, Philippines, *terroirs* known for their significant historical, cultural, and aesthetic values. However, heritage heirloom rice farming is gradually being abandoned, mainly because of its lower productivity and the struggle of the sector to create a sustainable niche market for heirloom rice by branding its cultural, social, and nutritional values. We propose several demand-side intervention strategies for the valorization of heirloom rice. To support the development of a segmented marketing strategy for heritage farming, we provide evidence on urban consumers’ willingness to purchase heirloom rice. We interviewed 500 urban consumers from Metro Manila in July–August 2015, who placed a purchasing bid on a kilogram of heirloom rice. Consumers’ bids averaged PHP 72.61 kg^−1^ (USD 1.60 kg^−1^), which is less than half its current market price. This explains why heirloom rice struggles to gain market share in urban markets in the Philippines. Given this bid price, we estimate a potential market size of PHP 20.3 billion (USD 443 million) that could be created for heirloom rice and tapped into by heritage farmers. Findings further indicate that women, business owners, and consumers who buy packaged rice and eat pigmented rice are willing to pay more for heirloom rice. Finally, our evidence suggests that proper information framing will be necessary to create demand and support the valorization of heirloom rice to preserve cultural heritage and in situ biodiversity of rice landraces in the Philippines.

## Introduction

Heirloom rice varieties (HRVs) have been grown for centuries by the ancestors of indigenous people in the Cordillera Autonomous Region (CAR) of the Philippines. It is estimated that the total area of terraces planted with HRVs in the CAR amounted to 11,692 hectares in 2014, producing a total of 34,747 metric tons (mt) of paddy rice at an average yield of 2.97 mt per hectare (DA 2016a, b). HRVs have been grown mostly for home consumption, which represents approximately 80% of the total rice produced from HRVs (IRRI 2015). Traditionally, these varieties are grown for social, cultural, and spiritual purposes, and thus it is called heritage rice farming. The HRVs are indigenous, are handed down by generations, and have exceptional flavor, texture, color, and nutritional values similar to other organic rice varieties (Samyor et al. 2017). Because of this added-value and higher production costs, and despite the generally lower milling quality, the current market price of HRVs is significantly higher than the price of well-milled rice (Table 5; IRRI 2015; Sajise et al. 2012). The HRVs and the rice terraces on which these are grown feature significant historic and aesthetic values. In 1995, these terraces were inscribed on the UNESCO World Heritage List. Thus, there is a positive externality bestowed on the local communities, attracting tourism and local businesses that result in better provision of rural livelihoods.

This heritage farming is, however, gradually being abandoned due to various constraints in the value chain (VC), on the supply as well as the demand side. First, HRVs feature higher production costs relative to modern rice varieties. The current production cost of HRV paddy is almost triple the cost of producing modern rice varieties (Table 1); of which labor costs represent nearly two-thirds. CAR’s topography and the use of terraces further contribute to the high labor cost. The second constraint on the supply side is the longer growing period of most HRVs (e.g., up to 240 days) (DA and IRRI 2016; Sajise et al. 2012; Soriano and Herath 2018). This hampers farmers’ cash flow and their ability to meet monetary obligations throughout the season (e.g., credit payments and household expenses). Third, the yield (productivity) of HRVs is almost half that of modern rice varieties (Tad-awan et al. 2015), and the cost of post-production activities (collection and transport) of HRVs is substantially higher than in the case of modern varieties (Table 1). However, the small acreage does not justify the implementation of a breeding program to improve the agronomic characteristics of HRVs. Moreover, any breeding efforts would bear the risk of losing some of the varieties’ key characteristics and authenticity that earns them the generic brand “heirloom rice” in the first place. Finally, heritage farming is being threatened by biotic and abiotic stresses such as droughts and insect pests (RICE Inc. 2012; Tad-awan et al. 2015). Although identified as constraints, these aforementioned factors contribute to the authentic characteristics of HRVs such as aroma, color, eating, and cooking qualities. Aroma, for instance, is best developed when rice is grown in cooler temperatures during maturity, such as the terraces in the CAR. The popular HRV *jekyot*, for example, is a slow-growing pigmented glutinous rice which grows best on irrigated terraces at an elevation of 700 meters above sea level (DA 2016a, b). This variety is cold-tolerant, similar to other HRVs, and drought-resistant.

**Table 1:** Comparison of yield and production cost of heirloom rice varieties (HRVs) in the Cordillera Autonomous Region versus high yielding rice varieties (HYVs) in the Philippines, 2013–2014

Items	Unit	HRVs in Cordillera Autonomous Region					HYVs
		Benguet	Ifugao	Kalinga	Mountain Province	Average	
Seed	PHP/ha	2900	5495	2672	2292	3551	3057
Organic fertilizer	PHP/ha	917	685	896	1059	861	9849
Machineries	PHP/ha	4296	6362	22,874	5400	9070	8760
Labor	PHP/ha	90,154	56,076	104,923	91,032	83,233	22,480
Post-production activities	PHP/ha	16,772	20,208	18,686	22,013	18,887	1986
Production cost of paddy (rough)	PHP/ha	115,039	88,825	150,051	121,796	115,602	46,132
Processing	PHP/ha	13,380	20,890	30,310	25,470	20,837	24,822
Production cost of milled rice	PHP/ha	128,419	109,715	180,361	147,266	136,440	70,954
Yield of milled rice	MT/ha	1.68	2.05	2.91	2.83	2.20	3.66
Cost of milled rice	PHP/mt	76,440	53,520	61,980	52,038	61,909	19,367
Cost of milled rice	USD/mt	1470	1029	1192	1001	1191	372

Sources for costing of modern rice varieties are from Bordey et al. (2016); costing of heirloom rice is from DA and IRRI (2016). The exchange rate during the time of the consumer survey (July–August 2015) was: USD 1.00 = PHP 45.70 (BSP 2019)

*PHP* Philippine peso, *mt* metric tons

On the demand side, heritage farming in the CAR faces three major constraints. First, rice VCs of HRVs are geographically short in the domestic market (Cuevas et al. 2017; DA and IRRI 2016; DA 2016a, b). HRVs are widely available within the CAR provinces but challenging to find in local and urban consumption zones in other regions in the country. HRVs are being promoted through high-end niche markets by avant-garde chefs, but the product distribution of HRVs in the mainstream retail channels is limited. Therefore, local consumers have limited access to HRVs. Although HRVs have reached the international market (dela Cruz 2019; Estigoy 2010; Featherstone 2010), export supply and demand have turned out to be unsustainable (Domoguen 2019). Secondly, Filipino consumers exhibit a marked preference for white rice (Custodio et al. 2016), while HRVs are mostly pigmented and unmilled (e.g., the bran layer is not entirely removed). Lastly, heritage rice farming is being disrupted due to various pressures such as the rapid growth of urbanization and industrialization, and the rise of tourism industries. For instance, cultivated land is being diverted to these industries, which adversely affects agro-biodiversity and the agricultural labor sector. These sectors are also pulling labor from agriculture, resulting in labor scarcity and higher rural agricultural wages.

Given the multiple challenges facing HRVs, VC upgrading strategies are imperative to improve the livelihood of HRV farmers and preserve the cultural heritage of heirloom rice. Enhancing farmers’ access to markets and improving the performance of VCs critically hinge on the identification of end-market opportunities (Demont and Ndour 2015; Demont and Rizzotto 2012; My et al. 2018). Even though the Philippine government has already initiated market-oriented policies to create a local niche and export market for heirloom rice through the Cordillera Regional Development Plans 2011–2016 and 2017–2022 (NEDA 2010, 2017), empirical evidence to link HRV farmers to the domestic market is insufficient to guide policy makers. The purpose of the paper is to provide empirical evidence from the demand side that can support these policies through the *valorization* of heritage rice farming in the CAR of the Philippines. More specifically, the paper aims at estimating consumers’ willingness to purchase heirloom rice, and identifying the factors that affect consumers’ purchase decisions.

## Valorization of heirloom rice: demand-side interventions

Valorization of heirloom rice can be supported through three demand-side interventions: “place branding,” “geographic indications (GIs),” and “product differentiation.” These interventions are expected to increase the demand for the product, and thus increase its market share and value, which eventually contributes to the preservation of cultural heritage and in situ biodiversity of rice landraces.^1^ Previous valorization studies of cultural heritage concur that actively maintaining a heritage site (adaptive re-use and conservation) will generate considerably higher social goods (in terms of monetary values) (e.g., Wright and Eppink 2016; Barrena et al. 2014; Choi et al. 2010; Bedate et al. 2004). Additionally, valorizing cultural heritage goods could offer economic and social opportunities for smallholders at heritage sites (Winkel et al. 2019). It also may lead to creating new jobs (Greffe 2004) as a result of the expansion of the tourism sector and, thus, overall territorial development. In this study, we focus on the valorization of heritage goods through demand-side interventions. Still, we recognize that the latter need to go hand-in-hand with simultaneous production interventions (e.g., improved management practices and higher-yielding varieties) and the concomitant development of labor markets.

First, we refer to “place branding” as embedding “physical geography and culture” in heirloom rice products. This concept is similar to what was previously theorized as “embeddedness,” “localness,” and “territoriality” of food in agri-food systems (e.g., Stone and Glover 2017; Bowen and Mutersbaugh 2013; Selfa and Qazi 2005; Dubois 2019; Trivette 2015). The hypothesis is that there are consumer segments that place a premium on heritage goods relative to conventional goods. Thus, their willingness to pay is higher for products embodying geographical and cultural attributes (Feldmann and Hamm 2015). We argue that branding “place and culture” (e.g., “local,” “terrace,” “heirloom,” “heritage,” and “sustainable”) for heirloom rice through proper information framings conveyed through labeling and packaging can be a crucial strategy in realizing many economic and environmental benefits. Note that, in the period 2005–2016, heirloom rice was marketed by the NGO Eighth Wonder and branded through pictures of rice terraces (Stone and Glover 2017). Approximately 27.5 metric tons of heirloom rice were sold annually, mostly to the United States, but after a decade, it ceased operations (https://www.heirloomrice.com). This export volume was too small to significantly increase market access of heirloom rice farmers. It did, however, highlight the weaknesses and inefficiencies in current heirloom rice VCs and farmer cooperatives and raise awareness of quality standards. Domestic markets for heirloom rice, on the other hand, currently remain largely untapped. Therefore, in this article, we argue that instead of focusing on export markets, value chain actors and policy makers should focus on expanding domestic markets for heirloom rice into major urban consumption zones first. Marketing strategies can target wealthy segments of the urban population and consumers who consume organic or pigmented rice.

Our survey data (described later) reveal that most of the surveyed consumers in Metro Manila are unaware of heirloom rice. One of the main reasons could be that people residing in the capital city are remote from the country’s rice terraces. Based on a meta-analysis of monetary valuation studies of cultural heritage, Wright and Eppink (2016) found that population density in the immediate area around heritage sites correlates with their value. In other words, demand for a heritage good or service is higher in areas with higher local population densities. Therefore, “place branding” could help consumers that are remote from heritage sites recognize and value heirloom rice and, hence, increase domestic demand for heirloom rice in the Philippines.

The second proposed intervention is to protect the national heirloom rice industry through Geographical Indications (GIs). A specialty rice industry can be affected due to incomplete competition either by the ethos of free trade or local industries that might take the opportunity of higher price blending with other varieties (Biénabe and Marie-Vivien 2017; Giraud 2008; Jena and Grote 2010). Therefore, GI certification could be provided by the proper authorities, confirming that heirloom rice originates from a specific geographical *terroir* and guaranteeing the property rights, product purity, and unique characteristics of the product. The legal protection of GIs is ensured under the Trade-Related Aspects of Intellectual Property Rights (TRIPs) Council of the World Trade Organization (WTO) (Jena and Grote 2010; Mulik and Crespi 2011). The governments of India, Thailand, and the United States have already issued GI tags for several Basmati and Jasmine rice varieties (e.g., Mulik and Crespi 2011; Ngokkuen and Grote 2012a, b; Jena and Grote 2012). Ngokkuen and Grote (2012a, b) noted that the adoption of GI certification by Thai farmers is low, and the adoption decisions are affected by social and human capital. Although past empirical studies suggested that consumers’ willingness to pay for GI tags and farmers’ adoption of GI certification has been limited in the case of rice (Seetisarn and Chiaravutthi 2011), a recent study among urban consumers in Thailand provides evidence that Bangkok consumers are willing to pay a premium for GI and protected GI-labeled Jasmine rice (Lee et al. 2020). The results of this study and similar studies on sustainability and traceability certification (e.g., My et al. 2018), further suggest that the provision of more detailed information about the GI labels can further increase consumers’ valuation by educating rice consumers on the meaning of these certifications. Therefore, to institutionalize GI tags for HRVs for valorizing the country’s heirloom rice industry, intervention by the Philippines government is needed. Currently, in the Philippines, Cordillera Heirloom Rice is among the products identified as potential GI products by the Intellectual Property Office of the Philippines (IPOPHL 2020). Two Philippine products (e.g., *Guimaras* mangoes and *Tau’ Sebu T’nalak*) have been registered as collective marks, as one of the legal mechanisms to protect a product through a GI. For heirloom rice, efforts towards GI registration have been made by the Department of Agriculture (DA) and the Department of Trade and Industry Intellectual Property Office (DTI-IPO), such as the development of Codes of Practice for selected HRVs, shelf-life testing, and development of the HRV Collective Mark (DA and IRRI 2017).

Finally, the concept of “product differentiation” is widely used as a marketing strategy for many commodities around the globe. There is ample evidence of growing consumer demand for rice quality attributes (Bairagi et al. 2020; Cuevas et al. 2016; Custodio et al. 2019; Demont et al. 2017; My et al. 2018). Therefore, by turning “localness” into a quality attribute, differentiation of heirloom rice from generic rice may help improve the economic sustainability of heirloom rice, thereby increasing social sustainability (e.g., by reducing emigration) and preserving cultural heritage in the Philippines. However, although heirloom rice has several quality attributes that set it apart from generic rice, such as flavor, aroma, and nutrition, it has never been valorized at a large scale (Stone and Glover 2017). More importantly, heirloom rice can substitute other organic or pigmented rice varieties that are available on the market. Therefore, it is crucial to investigate whether consumers are willing to pay price premiums for the quality attributes of heirloom rice.

## Materials and methods

### Economic model: consumers’ optimal bidding behavior

Suppose that consumers have private information about their preferences for both heirloom rice and other rice, denoted by $R_{h}$ and $R_{o}$, respectively, which are reservation prices for the product. If the consumer submits a bid $b$ to purchase the product, his or her utility will be $U\left( {R_{h} + b} \right)$,^2^ whereas, if the bid is not submitted, the consumer’s utility, $U\left( {R_{o} } \right)$, is the reservation utility. The consumer will tender a bid $b$ if the expected utility in case of consuming heirloom rice exceeds the reservation utility. Following Latacz-Lohmann and der Hamsvoort (1997), this bidding behavior can be mathematically expressed as:

1 $$U\left( {R_{h} + b} \right)\Pr \left( {b \le B} \right) + U\left( {R_{0} } \right)[1 - \Pr \left( {b \le B} \right)] > U\left( {R_{0} } \right)$$

where $Pr$ is the probability, and $B$ is the bidder’s expectations about the bid. It can be assumed that each bidder forms different expectations about *B*. Therefore, these expectations can be characterized by the density function $f\left( b \right)$ and cumulative distribution function $F\left( b \right)$. Then, using these density functions, the probability that the consumer submits a bid can be expressed as:

2 $$\Pr \left( {b \le B} \right) = \int\limits_{b}^{{\bar{B}}} {f\left( b \right)db} \approx 1 - F\left( b \right)$$

where $\bar{B}$ is the upper limit of the bidder’s expectations, such as the maximum expected bid. Substituting (2) into (1) yields the consumer’s optimal bidding problem:

3 $$U\left( {R_{h} + b} \right)\left[ {1 - F\left( b \right)} \right] + U\left( {R_{0} } \right)F\left( b \right) > U\left( {R_{0} } \right)$$

The left-hand side expression of Eq. (3) denotes the expected utility, whereas the right-hand side expression is the reservation utility. The consumer maximizes the expected utility over and above the reservation utility. If $b = 0$, then $U\left( {R_{h} } \right) = U\left( {R_{0} } \right)$, implying that consumer is indifferent between heirloom rice and other rice.

In our survey (see “Data” section), we bounded the values of $B$ between PHP 80 and PHP 130 per kilogram (kg^−1^), considering current market prices of substitute products (e.g., pigmented, and premium quality rice), with an interval of PHP 10.^3^ Therefore, respondents encounter six different bid prices. However, if the respondent does not have any intentions to purchase heirloom rice while he/she was asked to place the first bid, PHP 80 kg^−1^, then he/she was not further invited to bid. Similarly, if the maximum bid, PHP 130 kg^−1^, was placed, consumers were not invited to place any higher bid.^4^ Econometrically, this consumers’ bidding behavior or their willingness to purchase (WTP) can be estimated with a standard probit model, which is discussed below.

### Econometric model

Since we collected a simple dichotomous answer from the respondents, $y_{i} = 1$ if the *i*-th respondent answers yes, and $y_{i} = 0$ if the answer is no, given a question about bidding a previously determined price for heirloom rice, WTP can be modeled through the following linear function:

4 $$WTP_{i} \left( {{\mathbf{X}}_{i} ,\varepsilon_{i} } \right) = {\mathbf{X}}_{i} {\varvec{\upalpha}} + \varepsilon_{i}$$

where ${\mathbf{X}}_{i}$ is a vector of exogenous variables presented in Table 2, and ${\varvec{\upalpha}}$ is a vector of coefficients associated with these exogenous variables to be estimated; $\varepsilon_{i}$ is an error term.

**Table 2:** Descriptive statistics of the variables used for regression analysis. *Source* Authors’ calculations based on the stated-preference survey data

Name of the variables	Definition	Mean	SD
Gender	If the respondent is a female = 1, and male = 0	0.83	0.38
Age	Age of the respondent in years	44.30	10.50
Civil status	If the respondent is married = 1, and single/divorced = 0	0.78	0.42
Household size	Total family members that are aged 20–60 years	2.94	1.43
Occupation	If the respondent is a professional (e.g., teacher, doctor, and lawyer) or business owner = 1, otherwise = 0	0.08	0.27
Dummy for income group			
Upper-income	If the household belongs to AB socio-economic classification of the society = 1, otherwise = 0	0.07	0.26
Upper-middle income	If the household belongs to C1 socio-economic classification of the society = 1, otherwise = 0	0.17	0.38
Lower-middle income (base)	If the household belongs to C2 socio-economic classification of the society = 1, otherwise = 0	0.75	0.43
Purchased packaged rice	If the respondent purchases packaged rice = 1, and 0 = if unpackaged/loose rice	0.24	0.43
Consumed substitute of heirloom rice	If the respondent’s family members consumed brown and pigmented rice in the past year = 1, otherwise = 0	0.28	0.45
Positive attitudes toward heirloom rice	If the unaware respondent shows a positive attitude toward heirloom rice after providing information on attributes of heirloom rice = 1, and 0 = if neutral or negative attitudes are shown	0.91	0.28
Membership	If the respondent is a member or supporter of any cultural or environmental group = 1, otherwise = 0.	0.30	0.46

*SD* standard deviation

It is assumed that the respondent answers yes when his/her WTP is greater than the expected bid price, $B_{i}$, for example, $WTP_{i} > B_{i}$, which is assumed to diverge across respondents. Therefore, the probability of observing a positive response given the values of the exogenous variables can be specified as:

$$\begin{aligned} \Pr \left( {y_{i} = 1{|}{\mathbf{X}}_{i} } \right) & = \Pr \left( {WTP_{i} > B_{i} } \right) \\ & = \Pr \left( {{\mathbf{X}}_{i} {\varvec{\upalpha}} + \varepsilon_{i} > B_{i} } \right) \\ & = \Pr \left( {\varepsilon_{i} > B_{i} - {\mathbf{X}}_{i} {\varvec{\upalpha}}} \right) \\ \end{aligned}$$

If $\varepsilon_{i}$ is normally distributed, $\varepsilon_{i} \sim N\left( {0,\sigma^{2} \left( { = 1} \right)} \right)$, then we have the following specification:

5 $$\begin{aligned} \Pr \left( {y_{i} = 1{|}{\mathbf{X}}_{i} } \right) & = \Pr \left( {\varepsilon_{i} > \frac{{B_{i} - {\mathbf{X}}_{i} {\varvec{\upalpha}}}}{\sigma }} \right) \\ & = 1 - {\Phi }\left( {\frac{{B_{i} - {\mathbf{X}}_{i} {\varvec{\upalpha}}}}{\sigma }} \right) \\ \Pr \left( {y_{i} = 1{|}{\mathbf{X}}_{i} } \right) & = {\Phi }\left( {{\mathbf{X}}_{i} \frac{{\varvec{\upalpha}}}{\sigma } - B_{i} \frac{1}{\sigma }} \right) \\ \end{aligned}$$

where $\Phi$ is the standard cumulative normal. Equation (5) is very similar to the traditional probit model except for the additional explanatory variable, $B_{i}$. Including it as an explanatory variable, we have ${\hat{\mathbf{\beta }}}\left( { = \frac{{{\hat{\mathbf{\alpha }}}}}{{{\hat{\mathbf{\sigma }}}}}} \right)$ and ${\hat{\mathbf{\gamma }}} = - \frac{1}{{{\hat{\mathbf{\sigma }}}}}$, which are the vectors associated with each of the explanatory variables and the bid coefficient, respectively. Finally, the mean WTP following Krinsky and Robb (1986) can be estimated as:

6 $$E\left( {WTP} \right) = - \frac{{{\hat{\mathbf{\beta }}}_{i} {\bar{\mathbf{x}}}_{i} }}{{{\hat{\mathbf{\gamma }}}}}$$

where ${\bar{\mathbf{x}}}_{i}$ is the vector of mean values of explanatory variables, and ${\hat{\mathbf{\beta }}}_{i}$ represents the vector of estimated coefficients. We estimate Eq. (6) using STATA 15 and the code wtpcikr developed by Jeanty (2007).

### Data

#### Sampling design and data collection

We designed a stated-preference survey and interviewed 500 respondents from households in the middle and upper socio-economic classes (SEC) in Metro Manila, Philippines.^5^ SEC, an indicator of household’s affluence level, is a spectrum whereby a household will fall into one of the classes AB, C1, C2, D, E with AB as the most affluent. The SEC classification is based on a point system using different parameters such as neighborhood and house appearance, facilities in the house (e.g., electricity and running water, refrigerator and washing machine, and car ownership). As a proxy for household income, AB is considered as an upper-income group, C1 as upper-middle, C2 as lower-middle, and D and E as low-income groups. The selection of respondents was made using a multi-stage sampling procedure: (i) random selection of *barangays*—village-level administrative units in the Philippines—proportionately distributed based on population; (ii) random selection of households within the selected barangays; and (iii) random selection of respondents through a probability respondent grid using the last birthday method. This is a selection procedure wherein all the names and birthdays of household members within the age of 20 and 60 years were listed. Then, the household member who had the most recent birthday was considered for further screening. The final screening is the household member’s involvement in purchasing decision making of rice consumed at home. The fieldwork was conducted in Metro Manila during July–August 2015 using a predesigned structured questionnaire. The main module of this questionnaire was about consumers’ awareness and consumption of different types of rice, including heirloom rice, their willingness to purchase heirloom rice, and their knowledge and attitudes toward these rice varieties. The socio-economic profile and other relevant information were also included in the questionnaire.

#### Consumers’ intention-to-purchase heirloom rice

**Fig. 1:**
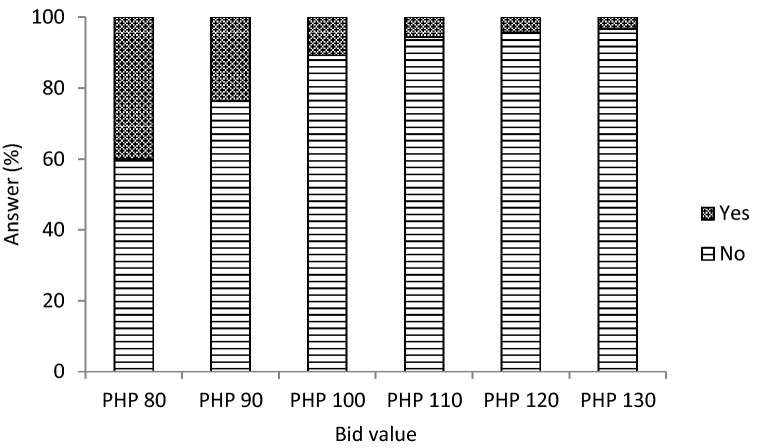
Consumers’ intention-to-purchase heirloom rice under different bid prices. *Notes* Authors’ estimations based on the stated-preference survey data. *PHP* Philippine peso. The exchange rate during the time of the consumer survey (July–August 2015) was: USD 1.00 = PHP 45.70 (BSP 2019)

Figure 1 illustrates consumers’ intention-to-purchase heirloom rice under the following six bid values, PHP 80, PHP 90, PHP 100, PHP 110, PHP 120, and PHP 130 kg^−1^ (USD 1.75–2.84 kg^−1^). Consumers were asked whether they would purchase heirloom rice at any of these bid values. We find that approximately 40% of consumers reported yes to the lowest bid price of PHP 80 kg^−1^, and as expected, their intention-to-purchase heirloom rice diminishes as price increases. For example, intention-to-purchase dropped to 10% at PHP 100 kg^−1^, and 3.4% at PHP 130 kg^−1^. This is consistent with the economic theory that demand decreases under increasing prices.

#### Consumers’ profiles

The sample respondents mostly comprise of married women (Table 2), which is not unusual since they are usually the grocery purchase decision-makers in Filipino households, and it is the main qualifying criterion for the survey. The average age of the sample respondents is 44 years; half of the sample is distributed in the age interval of 20–45, whereas the rest are distributed in the age interval of 46–60. The average family size, 3.0, is found to be comparatively smaller than the national average, which may be because we consider economically active persons aged 20–60 years living in the family. We also find that only 8% of the total sample of respondents were professionals and business owners. It is postulated that professionals and business owners are more concerned about a healthy lifestyle. As a result, their willingness to pay for organic and nutritious foods, such as pigmented heirloom rice, is likely to be higher. Pigmented rice is beneficial for health due to the presence of bioactive compounds in the bran (Samyor et al. 2017). The remaining respondents were either self-employed or unemployed (e.g., homemakers).

About social status, two-thirds of the surveyed respondents belong to the lower-income group (Table 2), which is not surprising as one-third of Filipino households are poor. The middle-income and upper-income groups represent 17% and 7% of the surveyed respondents, respectively. It is hypothesized that upper-income groups are the ones that are likely to demand specialty or premium rice.

Table 2 also shows that about one-third of the surveyed households have consumed brown and pigmented rice during the past year. This finding confirmed Filipino households’ occasional consumption of brown and pigmented rice, which is used for traditional dishes during different cultural occasions (Cuevas et al. 2017). Although the majority of the sample respondents (97%) were unaware of or never heard about heirloom rice, their attitude towards it favorably changed after providing them information on the attributes of heirloom rice (Table 2). About one-quarter of the respondents purchased packaged rice and supported cultural or environmental groups. These results may suggest that demonstrating the social and cultural values of heirloom rice through labeling and packaging (e.g., external quality cues) could be one of the possible demand interventions to create a local niche market for heirloom rice to preserve cultural heritage, theorized before.

## Results

### Determinants of consumers’ willingness to purchase

Table 3 presents the estimated parameters and the model diagnostic test statistics. We evaluate the goodness-of-fit of the model using the area under the Receiver Operating Characteristic (ROC) curve and Hosmer–Lemeshow (HL) statistics. The ROC curve evaluates whether the model is correctly classified, taking a value from 0 to 1, where 0 indicates the model has a perfectly inaccurate predictive power, and 1 reflects perfectly accurate predictive power.^6^ The HL test assesses whether the observed event rates match the expected event rates in the population subgroup; insignificant HL statistics suggests a satisfactory model fit. We find that the estimated ROC statistics is 0.794, implying the model’s excellent predictive power (Mandrekar 2010), and the estimated HL statistic is 9.38 and insignificant (last row of Table 3), implying that our model fits well. Therefore, below we provide economic ramifications of the estimated parameters, limiting our discussion only to marginal effects parameters.^7^

**Table 3:** Parameter estimates from the Probit model

Independent variables	Coefficient	Marginal effects
Bid amount	− 0.043*** (0.00)	− 0.008*** (0.00)
Gender (female = 1)	0.270*** (0.09)	0.057*** (0.02)
Age (years), log	0.13 (0.12)	0.028 (0.03)
Civil status (married = 1)	− 0.310*** (0.08)	− 0.064*** (0.02)
Household size (number), log	− 0.120* (0.06)	− 0.024* (0.01)
Occupation (professionals and business owners = 1)	0.300** (0.12)	0.061** (0.02)
Upper-income group (yes = 1) (base = lower-middle income group)	0.150 (0.13)	0.031 (0.03)
Upper-middle income group (yes = 1) (base = lower-middle income group)	0.050 (0.09)	0.010 (0.02)
Purchased packaged rice (yes = 1)	0.170** (0.08)	0.035** (0.02)
Consumed substitute of heirloom rice (brown and other pigmented rice = 1, otherwise = 0)	0.420*** (0.08)	0.086*** (0.02)
Positive attitudes toward heirloom rice after providing information (yes = 1)	0.470*** (0.13)	0.097*** (0.03)
Member of any social and environmental group (yes = 1)	− 0.037 (0.07)	− 0.008 (0.02)
Constant	2.160*** (0.55)	
Goodness-of-fit tests		
ROC statistics	0.794	
Hosmer–Lemeshow	9.38	

Dependent variable: 1 = yes to the WTP question

*** and ** denote statistical significance at 1% and 5% levels

Three major conclusions can be drawn from the results (Table 3): (i) consumers’ intention-to-purchase heirloom rice decreases significantly under increasing prices; (ii) the market segments that are more likely to pay higher price premiums for heirloom rice are women, business owners and professionals, consumers who already consume brown and pigmented rice and those who purchase packaged rice (rather than loose rice); and (iii) information matters as consumers’ intention-to-purchase heirloom rice increases after providing them with product information. Below, we discuss these findings in more detail.

Firstly, the marginal effect parameter for bid is − 0.008 and is significant at the 1% level (Table 3), indicating that the bid is negatively and significantly associated with buying intentions of heirloom rice. In other words, if the bid amount increases by PHP 10 kg^−1^, the probability of buying heirloom rice among those with positive buying intentions is likely to decrease by 8%. This finding suggests an inverse demand function consistent with economic theory. Moreover, it is consistent with studies conducted for ecosystem services (Barrena et al. 2014; Tadesse 2018), cultural heritage sites (Tuan and Navrud 2007, 2008) and demand for organic or sustainable food products (Klimas and Webb 2018).

Secondly, women were found to be 6% more likely to pay higher price premiums for heirloom rice than men. This contrasts with findings for regular rice in the Philippines, where no gender effect was found in any of the income classes with respect to willingness to pay for rice (Cuevas et al. 2016). Other consumer segments featuring a higher demand for heirloom rice include single consumers (6% higher likelihood to pay price premiums), professionals and business owners (6% higher likelihood), shoppers who buy packaged rice (4% higher likelihood) and consumers of brown and pigmented rice (9% higher likelihood). This finding is consistent with the fact that, as postulated before, high income and educated groups are more concerned about a healthy lifestyle (Divine and Lepisto 2005; McMorrow et al. 2017; Nakamura et al. 2016) and therefore are more likely to consume nutritious pigmented rice, pay attention to packages, and willing to pay more for heirloom rice. Moreover, most of the premium and organic rice on the market is packaged and features product (e.g., nutritional value) and cultural information (e.g., cultural heritage). Therefore, there is a need for market segmentation, targeting high-income and more educated households. Household size is negatively and significantly (10% level) associated with the probability of purchasing heirloom rice. This suggests that households are less likely to purchase expensive specialty or premium quality rice the more members it needs to feed.

Finally, we find that unaware shoppers who displayed positive attitudes toward heirloom rice after being exposed to information were 10% more likely to pay higher price premiums for the product than those who featured negative and neutral attitudes. This finding is consistent with studies on biofortified crops where it is generally found that consumers are willing to pay price premiums of 20% or more for these products when information on vitamin levels or health benefits is provided (see review by De Steur et al. 2017). Similarly, using experimental auctions, My et al. (2018) found that Vietnamese consumers’ willingness to pay for rice gradually increases when increasing levels of information are provided. Finally, using a randomized clinical trial among Chinese adults, Zhang et al. (2010) found a significant positive effect of information about brown rice on consumers’ willingness to consume brown rice. Therefore, we suggest that proper information framing could be a potential tool for making consumers aware of heirloom rice, and thus creating a niche market for it.

### Mean willingness to purchase

We use Eq. 6 to calculate the WTP for heirloom rice. The result shows that the mean WTP is around PHP 72.61 kg^−1^ (USD 1.60 kg^−1^) with a 95% confidence interval of PHP 69.38–75.27 kg^−1^ (USD 1.52–1.65 kg^−1^). This estimate is within the broad range of revealed prices that the surveyed consumers paid for domestically produced “super-premium” rice such as Dinorado- and Sinandomeng-labeled rice (PHP 58.24 kg^−1^), brown rice (PHP 77.50 kg^−1^) and pigmented rice (PHP 78.95 kg^−1^). However, the market price of heirloom rice is in the range of PHP 120–340 kg^−1^ and hence more than double the price of premium quality rice (Cuevas et al. 2018; Table 5). This may be the main reason why heirloom rice has been struggling to gain market share in urban markets in the Philippines.

### Potential market size for heirloom rice

In Table 4, we estimate the demand for heirloom rice in the Philippines. Findings indicate that local demand for heirloom rice could be up to 279,099 metric tons, equivalent to approximately PHP 20.3 billion (USD 443 million). To estimate the market size for heirloom rice, we consider the following steps. Firstly, we calculate the mean predicted probability from the Probit model across income groups. We find that the likelihood of purchasing heirloom rice is the highest for the upper-income group (25.1%), followed by the upper-middle-income group (18.6%), and the lower-middle-income group (15.6%). Secondly, we calculate the total number of families in the Philippines that consume special or premium rice based on the data reported in NSO (2010). We find that around 4.14 million families (about 20% of the total families) consume premium or specialty rice. On average, a Filipino family consumes around 330 kg of specialty rice annually (NSO 2010); however, an upper-income (wealthy) family consumes around 413 kg of specialty rice, followed by 339 kg by a middle-income and 267 kg by a lower-income family (Table 4). Thirdly, we multiply the total consumption of specialty rice by the probability of purchasing heirloom rice and find that local demand for heirloom rice could be between 237,939 and 279,099 metric tons annually in the Philippines. Finally, using the estimated mean willingness to purchase price, PHP 72.61 kg^−1^ (USD 1.60 kg^−1^), we calculate the market value of heirloom rice. The result indicates a potential market size of PHP 17.3–20.3 billion (USD 378–443 million) that could be created for HRVs and tapped into by HRV farmers.^8^

**Table 4:** Potential market size of heirloom rice in the Philippines

Income category	No. of families (in 000)^a^	Annual consumption of special rice (kg/family)^a^	Demand for special rice (metric tons)	Predicted probability of purchasing heirloom rice^b^			Demand for heirloom rice (metric tons)		
				Mean	[95% CI]		Mean	[95% CI]	
	a	b	c = a × b	d			e = c × d		
Upper-income group (10th decile)	971	413	259,396	0.251	0.223	0.278	100,657	89,428	111,484
Upper-middle income group (8th and 9th deciles)	1213	339	410,601	0.186	0.171	0.201	76,372	70,213	82,531
Lower-middle income group (bottom 70%)	1955	267	807,415	0.156	0.150	0.163	81,430	78,298	85,084
Total	4139						258,458	237,939	279,099

^a^Authors’ computation from NSO (2010)

^b^Estimated from Probit model

## Conclusions and policy recommendations

Valorization of heirloom rice through demand-side interventions is expected to increase the demand for the product, increase its market share and value, and consequently preserve cultural heritage and in situ biodiversity of rice landraces on the rice terrace *terroirs* in the Cordillera Mountains of Luzon, Philippines. To support the development of a segmented marketing strategy for heritage farming, we estimate urban Filipinos’ willingness to purchase heirloom rice and identify the factors that influence their purchase intentions. From the empirical analysis, it can be inferred that consumers’ willingness to pay for heirloom rice is in the range of the prices paid for premium rice, but lower than current prices of heirloom rice that are being sold in urban markets in the Philippines. Therefore, either demand for heirloom rice needs to be lifted by adding value to the product; or production costs need to be reduced through investments in enhancing productivity and value chain upgrading. Our study provides evidence that can help policy makers and value chain actors implement the first strategy.

First, policy makers and value chain actors should invest in information campaigns to educate and inform consumers about the social, cultural, environmental, and nutritional value of heirloom rice in the Philippines. The value and importance of preserving the Cordillera rice terraces and the cultural heritage that Filipinos host also need to be communicated to consumers. This is because our findings indicate that urban consumers are unaware of the values of heirloom rice varieties grown in the Cordillera region.

Secondly, a two-tier marketing strategy needs to be developed. On the one hand, the strategy should focus on retaining the market segments that are currently paying price premiums for heirloom rice, such as women, singles, professionals and business owners, brown and pigmented rice consumers and shoppers who purchase packaged rice. On the other hand, marketing should simultaneously focus on expanding market share to other market segments and target their advertising campaigns to men, married households, other professions, and premium white rice consumers. Previous evidence suggests that premium white rice consumers can be convinced to switch to heirloom rice if they are informed about the role they can play in preserving cultural heritage (Cuevas et al. 2018). In both cases (e.g., retention and expansion), the product’s distribution channels should be carefully considered. For the existing market segment, for instance, the promising channels of heirloom rice may include high-end retail outlets (e.g., for the upper-income group), convenient retail stores (e.g., for the professionals), and institutional establishments (e.g., for out-of-home consumption of single professionals). A different distribution strategy can be further explored to expand market share to other segments. For instance, mid-range retail outlets may be more visited by the broader middle class. Hence, including heirloom rice in the store’s product portfolio may potentially attract trial purchases from premium white-rice consumers. Based on our data, we estimate local demand for heirloom rice to be 237,939–279,099 metric tons, equivalent to a PHP 17.3–20.3 billion (USD 378–443 million) market value of heirloom rice in the Philippines.

Thirdly, it is vital to label the cultural and social value and value of sustainability of heirloom rice in the package, through terms such as “heirloom”, “heritage”, “organic”, and “fair trade” (Glover and Stone 2018). Several studies have confirmed that extrinsic quality cues, such as labels and packaging, play a critical role in reinforcing a product’s value and quality expectations (in the case of rice, see e.g., Custodio et al. 2019; Demont and Ndour 2015; Demont et al. 2013; My et al. 2018).

Finally, instead of focusing on rice alone, marketing research should study consumer preferences for heirloom rice and product positioning in a culture-specific “gastronomic system” to assess its suitability within rice-based diets (Cuevas et al. 2017). This research can reveal the occasions during which heirloom rice will be most likely consumed as well as the dishes that are most suitable for heirloom rice consumption and the ingredients that pair best with heirloom rice. The latter can help marketers better positioning the product, relative to its substitutes (e.g., brown rice, organic rice, and other pigmented rice). Celebrity chefs could play a major role in the development and promotion of new eating occasions, dishes and ingredient pairings based on heirloom rice. This information would be very much appealing and may be added in the packaging and/or as in-store merchandise to complement the cultural and social values of the heirloom rice with information, which can be considered as more practical—why, when, and how to consume heirloom rice.

Valorization of heritage rice farming will require substantial government intervention in the demand side through investment in public information campaigns and research, and by creating an enabling environment for crowding-in of private sector investment in advertising, branding, and upgrading of heirloom rice value chains. Our study provides some first insights into urban Filipino consumers’ response to such marketing strategies. Future research should expand this knowledge and generate evidence of consumers’ willingness to pay for place branding and GI labeling (e.g., Lee et al. 2020) of heirloom rice. Future research could also explore how a consumer experience of contributing to the preservation of cultural heritage (e.g., Cuevas et al. 2018) can be staged and embodied in the product. Promoting heritage products may risk increasing prices to the extent that local consumers can no longer afford them, though. This may put pressure on the production system and increase its environmental footprint. Moreover, due to low productivity and high seasonality of heirloom rice production, private sector investment in heirloom rice value chains is currently limited. Therefore, government intervention in the supply side is needed as well. The Philippines government already encourages and supports farmers in the Cordillera Mountains to organize themselves in cooperatives (e.g., the Rice Terraces Farmer Cooperative—RTFC in Banaue, Ifugao) and even subsidizes milling equipment. Apart from supply-side interventions, it is important that farmer cooperatives are also involved in the demand-side interventions, and preferably in an early stage, such that they can rapidly respond to increasing demand for heirloom rice from urban markets through supply of heirloom rice that meets the expectations of urban consumers. Failure to do so may result in unsatisfied and unsustainable demand (Demont and Rizzotto 2012). Therefore, government support needs to expand towards enhancing farmer cooperatives’ entrepreneurial and marketing capacity and supporting downstream integration of their business model towards milling, wholesale and retail. Simultaneous investment in demand-side and supply-side interventions is expected to catalyze crowding-in of additional private sector investment in heirloom rice value chains. Future research needs to focus on the optimal government interventions and conditions that can sustainably preserve rice cultural heritage and in situ biodiversity of rice landraces through the development of heirloom rice value chains in the Cordillera Mountains of Luzon, Philippines.

## Annex

See Table 5.

**Table 5:** Heirloom rice prices in the Philippines in January 2020

Heirloom rice variety	Selling price by cooperatives in CAR	Retail price	Retail channel
Balatinaw (black rice from Mountain Province)	PHP 120 kg^−1^	PHP 340 kg^−1^ Label: Holy Crop Organic Rice—Black	Online—Shopee https://shopee.ph/HOLY-CROP-ORGANIC-RICE-BLACK-1-KILO-i.31724887.435933452
		PHP 197.50 kg^−1^ Label: Prime Organics Premium Black Rice (2 kg pack, PHP395)	Online—Lazada https://www.lazada.com.ph/products/prime-organics-premium-black-rice-2kg-i257935641-s355408399.html?laz_trackid=2:mm_150020660_51201560_2010251560:clk5hhokh1du77a42em2gr
Chong-ak Unoy (red rice from Kalinga)	PHP 150 kg^−1^	PHP 230 kg^−1^ Label: Red Heirloom Rice, Unoy/Chong-ak	Online—Shopee https://shopee.ph/Red-Heirloom-Rice-Unoy-Chong-ak-i.82197908.1461973065
		PHP 150 kg^−1^ Label: Kalinga Unoy Heirloom Rice	Modern retailer based in Tabuk City, Kalinga
Ominio	n.a.	PHP 160 kg^−1^ Label: Heirloom Rice from the famed terraces of the Philippines—MOUNTAIN VIOLET (Sticky Rice)	Online—Shopee https://shopee.ph/Heirloom-Rice-MOUNTAIN-VIOLET-(Sticky-Rice)-i.70745408.1181163221
		PHP 258 kg^−1^ Label: Banaue Organic Purple Rice (5 kg, PHP 1290)	Online—Bigas PH https://www.bigasph.com/collections/heirloom-organic-rice/products/mountain-violet-sticky-rice-5kg
Minaangan (red rice)	n.a.	PHP 330 kg^−1^ Label: Hopy Crop Heirloom Rice—Red	Online- Shopee (Holy Crop!) https://shopee.ph/HOLY-CROP-HERILOOM-RICE-RED-1-KILO-i.31724887.435933507
		PHP 258 kg^−1^ Label: Banaue Organic Red Rice (5 kg, PHP 1290)	Online—Bigas PH https://www.bigasph.com/collections/heirloom-organic-rice/products/heirloom-organic-red-rice
		PHP 150 kg^−1^ Label: Heirloom Rice RED RICE (MINAANGAN)	Online—Shopee https://shopee.ph/Heirloom-Rice-RED-RICE-(MINAANGAN)-1kg-i.70745408.1181170238

The retail prices from online channels do not include the shipping fees. The exchange rate in January 2020 was US$1.00 = PHP50.86

*CAR* Cordillera Autonomous Region, *PHP* Philippines Peso

## Abbreviations

CAR: Cordillera Autonomous Region
DA: Department of Agriculture
GIs: Geographic indications
HRVs: Heirloom rice varieties
IRRI: International Rice Research Institute
NGO: Non-governmental Organization
PHP: Philippine peso
ROC: Receiver Operating Characteristic
SEC: Socio-economic classes
TRIPs: Trade-Related Aspects of Intellectual Property Rights
USD: United States Dollar
VC: Value chain
WTO: World Trade Organization
WTP: Willingness to pay/purchase

## Footnotes

1 Previously, Glover and Stone (2018) and Sekimoto and Augustin-Jean (2012) proposed to create a market for heirloom rice in the Philippines through the concept of *commodification*, which is quite similar to ours but distinct in many aspects, such as the fact that we primarily focus on demand-side interventions.

2 It is assumed that the utility function satisfies all the standard economic properties, including monotonicity and twice differentiable (Varian, 1992).

3 PHP stands for Philippine peso, the currency of the Philippines. The exchange rate during the time of the survey (July–August 2015) was: USD 1.00 = PHP 45.70 (BSP 2019).

4 The main problem of binding the maximum bid value is that $\bar{B}$ cannot be observed, for example, some consumers’ willingness-to-pay or their reservation utility could have been even higher than the bounded maximum bidding value.

5 Informed consent was obtained from the participants before starting the survey, and participants were informed that the data would be anonymized. Moreover, we followed all other standard data collection protocols. The full dataset is publicly available at Harvard Dataverse (Custodio and Demont 2020).

6 It also graphs sensitivity, the fraction of observed positive-outcome cases correctly classified, versus one minus specificity, the fraction of observed negative-outcome cases correctly classified.

7 Unlike estimated coefficients from the Probit model, marginal effects can inform both on the direction of the causal relationship of a predictor and its magnitude (Greene 2012).

8 Since we did not control for endogeneity and selection bias caused by some of the exogenous variables in the regression analysis, the estimated market demand should be used or interpreted with caution.
